# Effectiveness of a serious game addressing guideline adherence: cohort study with 1.5-year follow-up

**DOI:** 10.1186/s12909-021-02591-1

**Published:** 2021-03-30

**Authors:** Tobias Raupach, Insa de Temple, Angélina Middeke, Sven Anders, Caroline Morton, Nikolai Schuelper

**Affiliations:** 1 Department of Cardiology and Pneumology, Göttingen University Medical Centre, Robert-Koch-Straße 40, 37075 Göttingen, Germany; 2 Department of Medical Education, University Hospital Bonn, Venusberg-Campus 1, Gebäude 33, 53127 Bonn, Germany; 3 Department of Legal Medicine, University Medical Centre Hamburg-Eppendorf, Butenfeld 34, 22529 Hamburg, Germany; 4 Nuffield Department of Primary Care Health Sciences, Medical Sciences Division, University of Oxford, Radcliffe Primary Care Building, Radcliffe Observatory Quarter, Woodstock Road, Oxford, OX2 6GG UK; 5 Medius KLINIK Ostfildern-Ruit, Hedelfinger Straße 166, 73760 Ostfildern-Ruit, Germany

**Keywords:** Guideline, Emergency, Management, Digital, Simulation, Serious game

## Abstract

**Background:**

Patients presenting with acute shortness of breath and chest pain should be managed according to guideline recommendations. Serious games can be used to train clinical reasoning. However, only few studies have used outcomes beyond student satisfaction, and most of the published evidence is based on short-term follow-up. This study investigated the effectiveness of a digital simulation of an emergency ward regarding appropriate clinical decision-making.

**Methods:**

In this prospective trial that ran from summer 2017 to winter 2018/19 at Göttingen Medical University Centre, a total of 178 students enrolled in either the fourth or the fifth year of undergraduate medical education took six 90-min sessions of playing a serious game (‘training phase’) in which they managed virtual patients presenting with various conditions. Learning outcome was assessed by analysing log-files of in-game activity (including choice of diagnostic methods, differential diagnosis and treatment initiation) with regard to history taking and patient management in three virtual patient cases: Non-ST segment elevation myocardial infarction (NSTEMI), pulmonary embolism (PE) and hypertensive crisis. Fourth-year students were followed up for 1.5 years, and their final performance was compared to the performance of students who had never been exposed to the game but had otherwise taken the same five-year undergraduate course.

**Results:**

During the training phase, overall performance scores increased from 57.6 ± 1.1% to 65.5 ± 1.2% (*p* < 0.001; effect size 0.656). Performance remained stable over 1.5 years, and the final assessment revealed a strong impact of ever-exposure to the game on management scores (72.6 ± 1.2% vs. 63.5 ± 2.1%, *p* < 0.001; effect size 0.811). Pre-exposed students were more than twice as likely to correctly diagnose NSTEMI and PE and showed significantly greater adherence to guideline recommendations (e.g., troponin measurement and D-dimer testing in suspected PE).

**Conclusions:**

The considerable difference observed between previously exposed and unexposed students suggests a long-term effect of using the game although retention of specific virtual patient cases rather than general principles might partially account for this effect. Thus, the game may foster the implementation of guideline recommendations.

**Supplementary Information:**

The online version contains supplementary material available at 10.1186/s12909-021-02591-1.

## Introduction

Clinical practice guidelines are the mainstay of patient management. Amongst others, they are useful in identifying the cause of chest pain [1], shortness of breath [2] and headache [3]. The importance of adherence to guideline recommendations for patient outcomes has recently been demonstrated in an international registry study [4]. Given that these recommendations mainly relate to clinical reasoning and decisions that need to be taken at the bedside, hands-on training appears to be the most promising activity to help young physicians familiarise with guideline recommendations. While shadowing is an effective teaching method for more advanced students [5], there is no guarantee that medical students or residents will be exposed to a sufficient number of patients presenting with the respective symptoms and diseases. Students and young physicians should only be allowed to deal with such patients under close supervision by advanced clinicians in order to avoid putting patients at risk. At the same time, they need to take on an active role during learning as this is a prerequisite for successful training [6]. Given the need to improve guideline adherence [7], innovative teaching methods are needed.

An intriguing option is the creation of an environment allowing for greatest possible immersion in a real-world setting of patient care. In order to increase learner motivation, such environments are increasingly being created in the context of so-called serious games presenting specific medical content but also incorporating aspects of gamification (e.g., content unlocking, leaderboards and virtual goods) to various degrees [8]. While some research in this area has already been performed, published studies tend to include small student samples [9], suffer from confounding factors limiting their interpretation (e.g., self-selection of students interested in digital resources [10]; lack of a control group [11]), assess student satisfaction rather than learning outcome itself [12] and do not allow conclusions to be drawn on long-term effectiveness as data were collected directly after using a serious game [13]. The use of digital formats (including virtual environments [14] and gamified solutions [15]) has increased considerably during the first 3 months of 2020. While patient-centred teaching should neither be suspended altogether nor fully replaced by digital formats, high-quality online simulations may yield beneficial effects for the acquisition of competencies related to clinical reasoning.

The objectives of this longitudinal study with a follow-up period of 1.5 years were (i) to compare short-term learning outcome between medical student groups at different levels of clinical education, (ii) to assess retention of clinical reasoning competence and (iii) to investigate student performance with regard to guideline recommendations as a function of ever having been exposed to the game compared to no previous exposure.

## Methods

### Educational setting

This prospective cohort study [16] was conducted at Göttingen University Medical Centre. During the three-year clinical phase of the undergraduate medical curriculum, students take 21 consecutive modules and complete clinical attachments. Content related to cardiology and respiratory medicine is primarily taught in two modules lasting 6 weeks each – one in the fourth and one in the fifth year: At the beginning of the fourth year, all students take module 09 in which diagnosis and treatment of cardiac and pulmonary disorders are taught in a systematic manner, including lectures, seminars, small-group case-based learning, post-mortem demonstrations, auscultation training with a cardiopulmonary patient simulator, and bedside teaching. In the middle of the fifth year (i.e., 1.5 years after taking module 09), students participate in a repetition module (module 20). Here, content from all previous modules is revisited from a perspective of presenting complaints; symptoms and findings suggestive of cardiovascular disease are revised in the first week of the module.

### Intervention

In 2017, a digital teaching innovation rooted in self-determination theory [17] was introduced to the clinical curriculum at Göttingen University Medical Centre. Students enrolled in modules 09 and 20 were given the opportunity to play six 90-min sessions of a serious game simulating an accident & emergency (A&E) department. These six sessions (one per week) are referred to as the ‘training phase’. In the game, students take on the role of the attending physician and need to triage virtual patients as well as take a history, order laboratory and other diagnostic tests, make a diagnosis, initiate treatment, and transfer patients to the most appropriate care unit within the virtual hospital. Following the discharge of a patient, students can access a feedback screen indicating the correct diagnostic pathway and recommended treatments. We have recently demonstrated the non-inferiority of this approach compared to small-group problem-based learning [18]. A short demonstration of the game can be viewed in the online supplement (see additional file ‘Video 1’).


**Additional file 1: Video 1.** Demonstration of the serious game used in this study. Caption: This MP4 file presents a short screencast of the digital simulation. The basic functions are explained by the first author of the manuscript.

In each session, up to 50 students sitting in the institution’s computer resource unit were exposed to up to 8 virtual patients in order to force them to prioritise problems and deal with time pressure while making clinical decisions. Sessions were facilitated by experienced clinical teachers who were present in the institution’s computer resource unit and answered questions on patient management as they came up. Student activity during each session was recorded in an Excel file (‘log-file’) documenting each specific action (i.e., questions asked, laboratory tests ordered, diagnosis, treatment and transfer). In both modules, the first session (week 1) served as a training session for students to familiarise with technical aspects of playing the game. Virtual patient cases presented in weeks 2 and 6 were similar albeit not identical with different ages, symptoms and observations to avoid simple recognition, thus facilitating an assessment of the performance increase between the two sessions. The three specific virtual patient cases analysed were created by one of the authors experienced in emergency care (N.S.). Presenting complaints in these virtual patients were shortness of breath (pulmonary embolism, PE), chest pain (non ST segment elevation myocardial infarction, NSTEMI) and headache (hypertensive crisis), respectively. Some of the virtual patients shown in weeks 3–5 of the training phase also presented with these symptoms so students had the opportunity to practice the management of these presenting complaints.

### Study samples

**Fig. 1:**
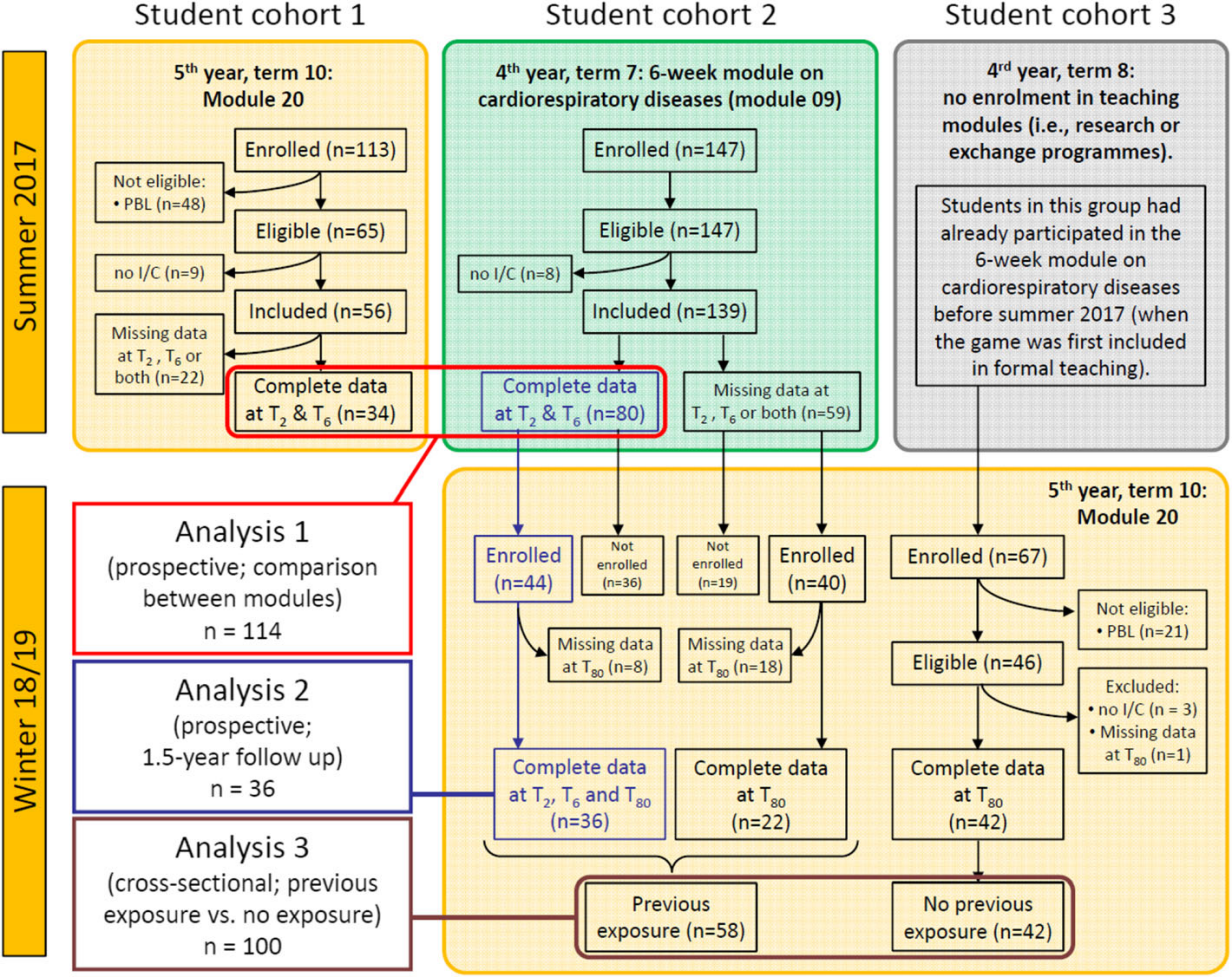
Flow of participants through the study and main analyses. Students were not eligible if they self-selected into the problem-based learning (PBL) group in module 20 or if they participated in a concomitant research project which would have contaminated the results of this study. I/C, informed consent; T_2_, week 2; T_6_, week 6; T_80_, week 80

Students enrolled in modules 20 (‘cohort 1’) and 09 (‘cohort 2’) in summer term 2017 were asked to provide written consent to have their game log-files analysed for research purposes. Students enrolled in module 20 in winter term 2018/19 (‘cohort 3’) were invited to play a session featuring the same three virtual patients described above. About half of these students had taken module 09 in summer term 2017; these students constituted the longitudinal 1.5-year follow-up cohort, and the session occurred in week 80 (winter term 2018/19) in relation to their original exposure to the game in the training phase. The remaining students had been enrolled in module 09 before the game was introduced to the formal curriculum (i.e., before summer term 2017). Since students could not access the game outside teaching sessions, these students had never been exposed to the game and the virtual patients presented in it. Traditionally, students enrolled in module 20 are given the opportunity to sign up for six 90-min sessions of problem-based learning (PBL). Since these sessions ran in parallel to serious game sessions, students opting into the PBL group were not eligible for study participation. In both modules, students were excluded from the analysis if they did not provide written consent or if there were missing data at any of the relevant time-points (see Fig. 1).

### Measurement of clinical reasoning performance

Clinical reasoning and guideline adherence was assessed by analysing log files of student activity while playing the serious game. Checklists were created for each of the three virtual patients with a respective maximum of 19 (chest pain), 14 (shortness of breath) and 10 (headache) points, thus producing a maximum overall score of 43. Total scores per virtual patient case were broken down in history and management sub-scores (see additional file ‘Online Supplement’; chapter ‘Methods – eTables 1a-c: Scoring sheets for log file analyses and proportions of students performing respective actions while using the game’). In order to increase comparability across virtual patient case scores, raw point scores were turned into percentages.

As part of the game, students were able to transfer or discharge patients, and these activities were also captured in the log files. Transfer decisions were not included in the checklists but were included in descriptive analyses.

### Statistical analysis

Following descriptive analyses and an assessment of the reliability of the scoring approach by means of Cronbach’s α [19] for each time-point, specific statistical tests were performed for each of the three study aims:

- Analysis 1: In order to address study aim (i), the difference in overall scores as well as history and management sub-scores from week 2 to week 6 was compared between students enrolled in the two modules by means of separate repeated measures ANOVAs. Proportions of correct diagnoses per virtual patient case were compared between week 2 and week 6 by McNemar tests.
- Analysis 2: In order to address study aim (ii), overall, history and management scores across the three virtual patient cases were compared across weeks 2, 6 and 80 in the longitudinal cohort by a Friedman test. In case of a significant result, separate paired T tests were performed to assess differences between the three data collection points.
- Analysis 3: In order to address study aim (iii), the three aforementioned scores were compared by independent T tests between students with previous exposure to the game and previously unexposed students. The impact of previous exposure on diagnostic reasoning was assessed in separate univariate logistic regressions for each virtual patient case.

Methods were carried out in accordance with relevant guidelines and regulations. Data are reported as mean ± standard error of the mean (SEM), proportions, and odds ratios (95% confidence interval) as appropriate. For significant differences, effect sizes are reported as Cohen’s d. Analyses were done with SPSS version 25 (IBM Corp., Armonk, New York, USA). Significance levels were set to 5%. This study was approved by the local Ethics Committee (Ethik-Kommission der Universitätsmedizin Göttingen, application number 15/3/17), and informed consent was obtained from all subjects.

## Results

### Student characteristics

Informed consent was obtained from all subjects. A total of 238 students provided written consent to participate in the study (overall response rate 92.2%). Due to missing data at any of the relevant time-points, only data from 178 students (74.8%) were included in the three main analyses (analysis 1: *n* = 34; analysis 2: *n* = 80; analysis 3: *n* = 100 with an overlap of 36 students between cohorts 2 and 3, representing the longitudinal cohort for study aim (ii)). The flow of participants through the study is illustrated in Fig. 1. Students were aged 25.3 ± 0.4, 24.9 ± 0.6 and 26.3 ± 0.4 years for analyses 1, 2 and 3, respectively. The proportion of female students was 62.3, 50.0 and 61.0%, respectively. With respect to analysis 3, a comparison of demographic data between students in cohort 2 (previous exposure) and cohort 3 (no previous exposure to the game) yielded no significant differences regarding age (26.8 ± 0.7 years vs. 25.6 ± 0.4 years; *p* = 0.152) or the proportion of female students (60.3% vs. 61.9%; *p* = 0.875).

Reliability analysis of the scoring approach yielded a Cronbach’s α of 0.669 (week 2), 0.771 (week 6) and 0.543 (week 80), respectively.

### Analysis 1: comparison between modules (prospective)

There was a significant and meaningful increase in student performance between week 2 and week 6 with no significant difference between the two modules at either time point (see additional file ‘Online Supplement’; chapter ‘Results – eFigure 1: Change in total, history and management scores from week 2 to week 6 in both modules (data collection in summer term 2017)’). Thus, all subsequent analyses were done on the combined sample of cohorts 1 and 2. Overall scores increased from 57.6 ± 1.1% to 65.5 ± 1.2% (*p* < 0.001; d = 0.656). The sub-score relating to history taking remained largely unchanged (from 57.9 ± 1.6% to 59.6 ± 1.6%; *p* = 0.329) while there was a substantial increase in the management sub-score (from 57.4 ± 1.2% to 69.8 ± 1.3%; *p* < 0.001; d = 0.919). This pattern of results was found for each of the three virtual patient cases (Table 1). Across the two modules, the proportion of students identifying the correct diagnosis increased from 57.9 to 80.7% for the virtual patient presenting with shortness of breath (*p* < 0.001), from 50.0 to 66.3% for the virtual patient presenting with chest pain (*p* = 0.014) and from 80.7 to 94.3% for the virtual patient presenting with a headache (*p* = 0.002).

**Table 1:** Change in history and management scores (mean ± SEM) for each virtual patient case between weeks 2 and 6. Due to the absence of a group difference, data from the two student cohorts were combined. *p* values were derived from paired T tests

Variable		Week 2	Week 6	***p*** value	Cohen’s d
Stable pulmonary embolism	History percent score	70.5 ± 1.9	70.5 ± 2.0	1.000	–
	Management percent score	53.9 ± 1.9	73.8 ± 1.7	< 0.001	1.02
Hypertensive crisis	History percent score	55.7 ± 2.4	58.2 ± 2.2	0.343	–
	Management percent score	57.4 ± 2.5	64.8 ± 2.2	0.007	0.34
NSTEMI	History percent score	46.3 ± 2.6	50.5 ± 2.3	0.110	–
	Management percent score	60.6 ± 1.9	69.0 ± 2.0	< 0.001	0.44

Transfer decisions taken by students were assessed by restricting the sample to only those students who had completed a specific virtual patient case at weeks 2 and 6. Results indicated that student abilities to decide where patient care should be continued increased over time (Table 2). For all three virtual patient cases, decisions tended to shift from a more intensive to a less intensive ward, indicating that student decisions became more appropriate and more resource-efficient.

**Table 2:** Transfer decisions of students identifying the correct diagnosis and transferring the respective patients from A&E at weeks 2 and 6, respectively. Data are presented as percentage (n)

Shortness of breath / Stable pulmonary embolism (*n* = 51)		
Transfer	**Week 2**	**Week 6**
discharge	0.0 (0)	2.0 (1)
normal ward	11.8 (6)	19.6 (10)
intermediate care unit	35.3 (18)	58.8 (30)
intensive care unit	49.0 (25)	17.6 (9)
operation theatre	3.9 (2)	2.0 (1)
Headache / Hypertensive crisis (n = 58)		
Transfer	**Week 2**	**Week 6**
discharge	19.0 (11)	75.9 (44)
normal ward	56.9 (33)	22.4 (13)
intermediate care unit	20.7 (12)	1.7 (1)
intensive care unit	3.4 (2)	0.0 (0)
operation theatre	0.0 (0)	0.0 (0)
Chest pain / NSTEMI (*n* = 22)		
Transfer	**Week 2**	**Week 6**
normal ward	13.6 (3)	27.3 (6)
intermediate care unit	54.5 (12)	68.2 (15)
intensive care unit	31.8 (7)	4.5 (1)

### Analysis 2: long-term effectiveness

**Fig. 2:**
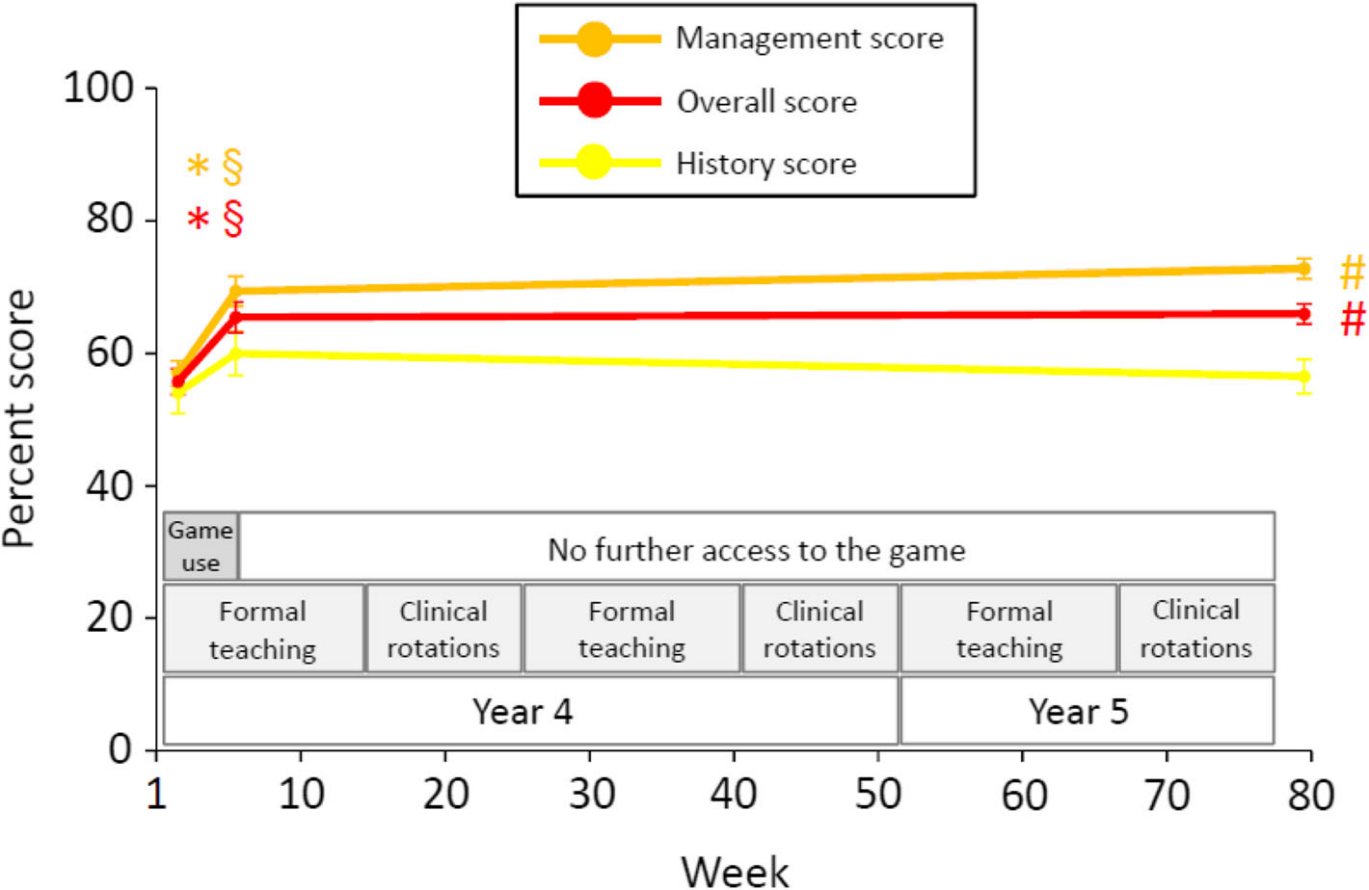
Change over time in total, history and management scores across virtual patient cases in the 1.5-year longitudinal cohort. Error bars indicate standard errors of the mean. #, p for Friedman test < 0.05; *, p for paired T test between week 2 and week 6 < 0.05; §, p for paired T test between week 2 and week 80 < 0.05

Analysis 2 revealed a sustained learning outcome for students who were followed up for 1.5 years (Fig. 2). After an initial increase in overall scores during the training phase, performance remained unchanged until students entered module 20. Again, analyses of sub-scores in the entire follow-up cohort showed a significant difference in management but not history scores. The observed pattern of results was confirmed for two virtual patient cases (shortness of breath / pulmonary embolism and chest pain / NSTEMI) while the Friedman test yielded non-significant results for the virtual patient presenting with a headache.

### Analysis 3: impact of ever-exposure versus non-exposure

**Fig. 3:**
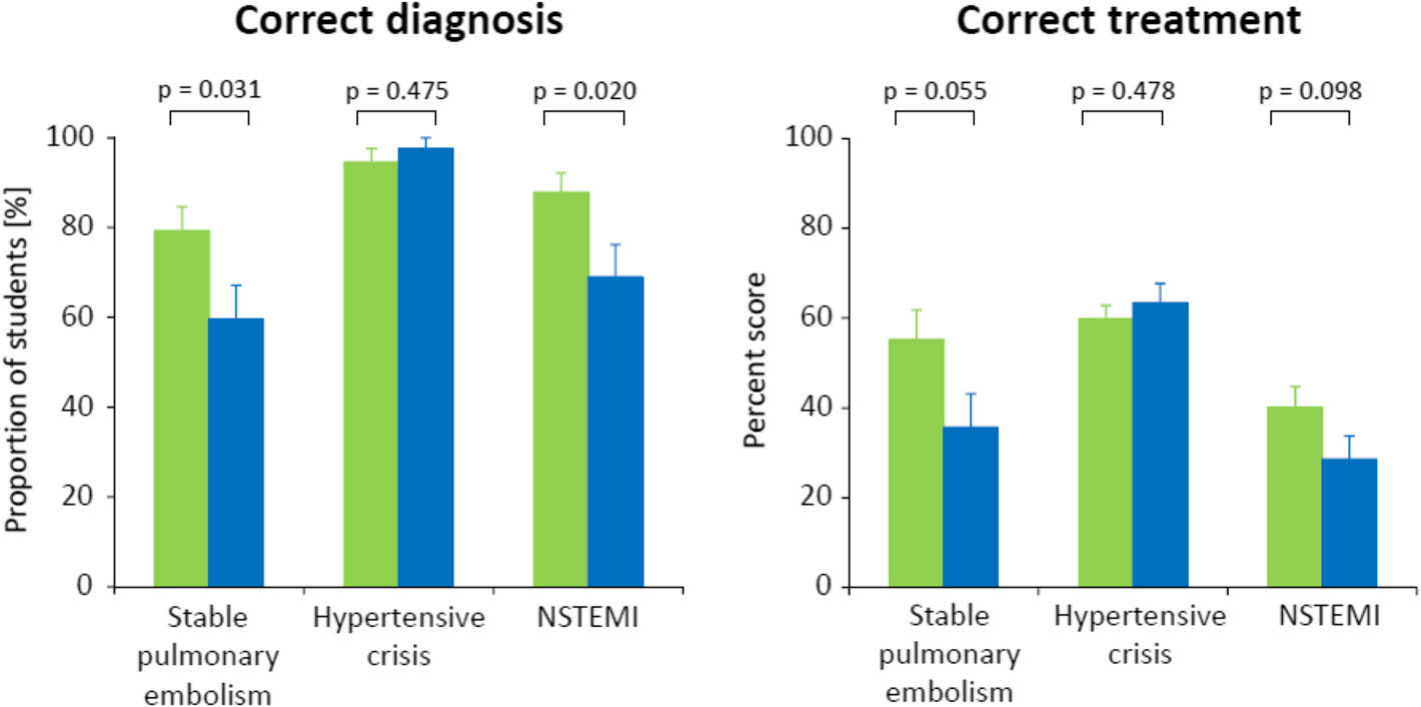
Differences in student performance per virtual patient case regarding diagnosis and treatment between students with (green; *n* = 58) and without (blue; *n* = 42) previous exposure to the serious game. Error bars indicate standard errors of the mean. *p* values were derived from χ^2^ tests (diagnosis, dichotomous) and independent T tests (treatment), respectively

In order to assess the impact of previous exposure to the game on clinical reasoning skills, students who had taken the training phase in module 09 were compared to students without previous exposure in winter term 2018/19 (analysis 3). The results presented in Table 3 indicate that performance of students with previous exposure was significantly better than performance of students with no prior exposure (d = 0.618). This effect was pronounced for items related to patient management (d = 0.811) and absent for items related to history taking (analyses by item are presented in the online supplement (chapter ‘Methods – eTables 1a-c: Scoring sheets for log file analyses and proportions of students performing respective actions while using the game’). As is shown in Fig. 3, previous exposure was associated with better diagnostic and therapeutic reasoning. Students who had played the game 1.5 years ago were more likely to correctly identify a stable pulmonary embolism (OR = 2.6; 95% confidence interval: 1.1–6.3) and an NSTEMI (OR = 3.3; 1.2–9.1). Differences were non-significant for treatment decisions.

**Table 3:** Differences in scores (mean ± SEM) between students with and without previous exposure to the serious game. Data were collected at week 80. *P* values were derived from independent T tests

Variable		Previous exposure (***n*** = 58)	No previous exposure (***n*** = 42)	***p*** value	Cohen’s d
All virtual patient cases combined	Overall percent score	65.6 ± 1.2	60.1 ± 1.4	0.003	0.618
	History percent score	55.9 ± 1.9	55.4 ± 2.1	0.875	–
	Management percent score	72.6 ± 1.2	63.5 ± 2.1	< 0.001	0.811
Stable pulmonary embolism	History percent score	63.2 ± 2.6	63.9 ± 3.6	0.878	–
	Management percent score	73.7 ± 2.2	58.0 ± 3.5	< 0.001	0.808
Hypertensive crisis	History percent score	61.6 ± 3.2	63.4 ± 3.5	0.709	–
	Management percent score	68.5 ± 2.1	69.5 ± 3.1	0.769	–
NSTEMI	History percent score	47.6 ± 2.3	44.9 ± 3.1	0.479	–
	Management percent score	74.0 ± 1.8	64.3 ± 2.2	0.001	0.713

## Discussion

It is important for physicians to be familiar with all aspects related to digital medicine. In fact, comprehensive learning resources for undergraduate as well as graduate medical education are already available in digital format but only very few high-quality studies addressing their effectiveness in terms of learning outcome have been published [20]. Among the challenges brought about by the coronavirus pandemic is the need to move a substantial part of medical education from lecture theatres and patient bedsides to the student home [21–23]. Against this background, evidence on the effectiveness of available approaches would be particularly useful.

Clinical practice guidelines are the mainstay of patient management and should be an integral component of undergraduate and postgraduate training. While factual knowledge on risk factors, diagnostic tests and treatment options may be learned from static resources (e.g. academic publications and books), clinical reasoning can best be trained by repeated exposure to real patient cases [24]. It has been suggested that e-learning can be an effective tool for implementing clinical practice guidelines [25], and a multicentre clinical trial testing this hypothesis is currently underway [26].

The present study provides first-time evidence of a sustained effect of using a serious game to train the management of patients presenting with symptoms suggestive of cardiovascular disease in accordance with current guidelines. Exposure to the game was associated with better diagnostic and therapeutic reasoning in the short- as well as in the long term: At the 80-week follow-up, students with previous exposure were twice as likely to correctly diagnose a patient with pulmonary embolism and three times as likely to correctly identify a diagnosis of NSTEMI. A similar pattern of results was observed for all three diseases assessed, suggesting a generalizable effect. Changes in overall scores following game use were driven by changes in management scores, indicating that the digital simulation fosters clinical reasoning while it does not have an effect on history taking skills. This might be due to the fact that taking a history from virtual patients involved selecting specific questions from a pre-defined list of 70 items which does not resemble clinical practice. Transfer decisions changed during the training phase, indicating that following game use, students took a more resource-sparing approach to patient management.

Figure 3 reveals that, irrespective of previous exposure to the game, student performance on diagnostic reasoning was substantially better than on treatment decisions. This finding is suggestive of a good construct validity of the analytic approach taken in this study as undergraduate medical education tends to focus on diagnostic rather than therapeutic reasoning.

### Impact of initial performance level on benefits elicited by the simulation

The similarity of observations in the two teaching modules in analysis 1 is surprising given the difference between the two modules: While students are introduced to cardiovascular medicine in year four (module 09), they are expected to deepen their knowledge over the course of the academic year and apply it from a perspective of presenting complaints in year five (module 20). We thus expected different levels of knowledge in student cohorts 1 and 2 at T2. In contrast, we found no difference in initial performance levels between the two student groups in a cross-sectional comparison. Most importantly, the data presented in eFigure 1 (no between-group differences at any time and similar differences between T2 and T6 in both groups) suggest that students benefitted from using the game equally and independently of initial performance levels.

### Potential of serious games to increase guideline adherence

The principle findings of this proof-of-concept study are that learning outcome elicited by a digital simulation of an A&E department is comparable in student cohorts in two different years of undergraduate education and that management skills acquired through the game were retained over a relatively long period of time during which it would otherwise have been lost in undergraduate education. This is important as poor retention of knowledge [27] and skills [28] has been demonstrated in medical students. If the present findings of favourable retention of management skills following game use also hold true for postgraduate medical education, the game may help increase adherence to guidelines in clinical medicine.

It has previously been shown that simulations can improve clinical performance of surgeons [29]. In addition, simulation-based education is most effective when errors are allowed to occur and can be used as starting points for further learning [30]. Digital resources have been used to improve diagnostic skills in undergraduate medical students [31] as well as prescribing knowledge among physicians [32]. In fact, online multimedia activities for continuing medical education have been shown to yield better outcomes than more traditional formats [33].

In terms of resources, it is worth considering the economic costs of serious games over live simulation training, which is often prohibitively expensive. Whilst games are costly to create, they require very little money to run and are not limited by numbers, which is the key drawback of live simulation training. Furthermore clinical reasoning and patient management skills are crucial for decision making in daily routine. Especially young doctors often struggle at this point as it is seldom addressed in undergraduate curricula. Serious games could help encourage and train students as well as young doctors to improve those skills enabling them to work in a more cost- and time-efficient way. Accordingly, a serious game like the one used in this study should not only be offered to students as it might also convey benefits for continuing medical education.

### Strengths and limitations

The main advantage of this study being situated within a medical school curriculum as opposed to a learning laboratory is that the effects of using the game in addition to formal training can be observed directly. Yet, the learning environment was more controlled than in postgraduate education where most activities are self-directed and not necessarily standardised. However, the approach taken in this study entailed specific limitations, including the lack of a clearly defined control group. All students enrolled in module 09 used the simulation. It would not have been feasible to create a credible control intervention.

While the observed increases in management sub-scores were significant and meaningful, student performance at week 80 was at best moderate (72.6 ± 1.2% in the intervention group). This might have been due to the fact that despite attendance at gaming sessions being mandatory, data collections were not linked to a summative (i.e., graded) examination. Previous research has shown that assessment format impacts considerably on student performance [34]. However, recent findings rooted in educational psychology suggest that repeated formative (i.e. non-graded) testing might have a more favourable effect on sustained learning outcome [35].

Performance scores were derived from student activity while playing the game. This measure is objective (i.e., it is independent of session facilitators), and reliable (favourable Cronbach’s α), and its face validity is apparent from the checklists provided (see additional file 'Online Supplement'; chapter ‘Methods – eTables 1a-c: Scoring sheets for log file analyses and proportions of students performing respective actions while using the game’). However, criterion validity of this outcome measure should be confirmed using a gold standard external criterion such as an objective structured clinical examination. Finally, student activities were confined to the virtual environment of the serious game. Further studies need to assess whether the observed effects translate into clinical practice.

## Conclusions

In this prospective trial with 1.5-year follow-up, the use of a virtual A&E department was associated with a sustained increase in performance levels regarding clinical reasoning. Results indicate that the game may foster the implementation of guideline recommendations. It may be particularly useful in times of self-isolation when medical education needs to be partially moved from lecture theatres to the student home.

## Supplementary Information

**Additional file 2:** Online Supplement. eTables 1a-c and eFigure 1.

## Data Availability

The datasets generated and/or analysed during the current study are not publicly available due this type of use not being included in the written consent form but are available from the corresponding author on reasonable request.

## Abbreviations

A&E: Accident and emergency
NSTEMI: Non ST segment elevation myocardial infarction
OR: Odds ratio
PE: Pulmonary embolism
SEM: Standard error of the mean
