# Supplementary material for: Effectiveness of a serious game addressing guideline adherence: cohort study with 1.5-year follow-up
Source: BMC Med Educ. 2021 Mar 30;21:189. doi: 10.1186/s12909-021-02591-1 (PMC8008024; doi:10.1186/s12909-021-02591-1)
Supplement: Supplementary file 2 — Additional file 2: Online Supplement. eTables 1a-c and eFigure 1. [file 12909_2021_2591_MOESM2_ESM.docx]

Online supplement

**Effectiveness of a serious game addressing guideline adherence:**

**Cohort study with 1.5-year follow-up**

Tobias Raupach, MD. MME^1,2^: raupach@med.uni-goettingen.de

Insa de Temple^1^: Insa.Frisch@gmx.de

Angélina Middeke, MD^1^: angelina.middeke@med.uni-goettingen.de

Sven Anders^3^: s.anders@uke.de

Caroline Morton^4^: caroline.morton@phc.ox.ac.uk

Nikolai Schuelper, MD^5^: n.schuelper@medius-kliniken.de

^1^ Department of Cardiology and Pneumology, Göttingen University Medical Centre, Robert-Koch-Straße 40, Göttingen, D-37075, Germany

^2^ Department of Medical Education, University Hospital Bonn, Venusberg-Campus 1, Gebäude 33, 53127 Bonn

^3^ Department of Legal Medicine, University Medical Centre Hamburg-Eppendorf, Butenfeld 34, Hamburg, D-22529, Germany

^4^ Nuffield Department of Primary Care Health Sciences, Medical Sciences Division, University of Oxford, Radcliffe Primary Care Building, Radcliffe Observatory Quarter, Woodstock Road, Oxford OX2 6GG, United Kingdom

^5^ Medius Klinik Ostfildern Ruit, Hedelfinger Straße 166, D-73760 Ostfildern-Ruit, Germany

**Methods – eTables 1a-c:** Scoring sheets for log file analyses and proportions of students performing respective actions while using the game 2

**Results – eFigure 1**: Change in total, history and management scores from week 2 to week 6 in both modules (data collection in summer term 2017).. 5

eTables 1a-c: Scoring sheets for log file analyses and proportions of students performing respective actions while using the game (comparison between pre-exposed and non-exposed students at week 80; analysis 3). Data are presented as percentage (n) or mean ± standard error of the mean, respectively. p values were derived from χ^2^ tests (dichotomous items) and independent T tests (sum scores), as appropriate.

**eTable 1a: Stable pulmonary embolism**

| **Stable pulmonary embolism** | **Previous exposure (n = 58)** | **No previous exposure (n = 42)** | **p value** |
| --- | --- | --- | --- |
| **History** | | | |
| Cough | 32.8 (19) | 26.2 (11) | 0.479 |
| Pain | 62.1 (36) | 52.4 (22) | 0.333 |
| History of presenting complaint | 70.7 (41) | 78.6 (33) | 0.375 |
| Smoking | 50.0 (29) | 54.8 (23) | 0.638 |
| Past medical history | 84.5 (49) | 95.2 (40) | 0.090 |
| Drug history | 79.3 (46) | 76.2 (32) | 0.710 |
| History: max. 6 points | 3.8 ± 0.2 | 3.8 ± 0.2 | 0.878 |
| **Management** | | | |
| Arterial blood gases | 87.9 (51) | 85.7 (36) | 0.745 |
| Troponin | 91.4 (53) | 71.4 (30) | 0.009 |
| D dimers | 79.3 (46) | 57.1 (24) | 0.017 |
| Electrocardiogram | 98.3 (57) | 83.3 (35) | 0.007 |
| Cardiac ultrasound | 29.3 (17) | 19.0 (8) | 0.242 |
| CT scan of the thorax | 69.0 (40) | 52.4 (22) | 0.092 |
| Correct diagnosis | 79.3 (46) | 59.5 (25) | 0.031 |
| Treatment: anticoagulation | 55.2 (32) | 35.7 (15) | 0.054 |
| Management: max. 8 points | 5.9 ± 0.2 | 4.6 ± 0.3 | <0.001 |
| Total score: max. 14 points | 9.7 ± 0.2 | 8.5 ± 0.3 | 0.003 |

**eTable 1b: Hypertensive crisis**

| **Hypertensive crisis** | **Previous exposure (n = 56)** | **No previous exposure (n = 41)** | **p value** |
| --- | --- | --- | --- |
| **History** | | | |
| Dizziness | 19.6 (11) | 17.1 (7) | 0.748 |
| History of presenting complaint | 66.1 (37) | 65.9 (27) | 0.982 |
| Past medical history | 71.4 (40) | 80.5 (33) | 0.307 |
| Drug history | 89.3 (50) | 90.2 (37) | 0.878 |
| History: max. 4 points | 2.4 ± 0.1 | 2.5 ± 0.1 | 0.641 |
| **Management** | | | |
| Full physical examination | 73.2 (41) | 73.2 (30) | 0.996 |
| Full blood count | 66.1 (37) | 70.7 (29) | 0.627 |
| Electrocardiogram | 57.1 (32) | 48.8 (20) | 0.415 |
| Correct diagnosis | 94.6 (53) | 97.6 (40) | 0.475 |
| Treatment: nitrates | 60.7 (34) | 56.1 (23) | 0.648 |
| Treatment: antihypertensive drugs | 58.9 (33) | 70.7 (29) | 0.232 |
| Management: max. 6 points | 4.0 ± 0.2 | 4.1 ± 0.2 | 0.678 |
| Total score: max. 10 points | 6.3 ± 0.2 | 6.6 ± 0.3 | 0.578 |

**eTable 1c: NSTEMI**

| **NSTEMI** | **Previous exposure (n = 58)** | **No previous exposure (n = 42)** | **p value** |
| --- | --- | --- | --- |
| **History** | | | |
| Difficulty breathing | 43.1 (25) | 42.9 (18) | 0.980 |
| Onset of presenting complaint | 55.2 (32) | 61.9 (26) | 0.501 |
| History of presenting complaint | 32.8 (19) | 16.7 (7) | 0.070 |
| Smoking | 36.2 (21) | 28.6 (12) | 0.423 |
| Allergies | 36.2 (21) | 26.2 (11) | 0.289 |
| Past medical history | 69.0 (40) | 71.4 (30) | 0.791 |
| Family history | 29.3 (17) | 40.5 (17) | 0.245 |
| Drug history | 79.3 (46) | 71.4 (30) | 0.362 |
| History: max. 8 points | 3.8 ± 0.2 | 3.6 ± 0.2 | 0.479 |
| **Management** | | | |
| Physical examination: thorax | 94.8 (55) | 88.1 (37) | 0.221 |
| Troponin | 100.0 (58) | 100.0 (42) | — |
| CK | 96.6 (56) | 92.9 (39) | 0.403 |
| CKMB | 98.3 (57) | 92.9 (39) | 0.172 |
| TSH | 27.6 (16) | 2.4 (1) | 0.001 |
| Electrocardiogram | 98.3 (57) | 97.6 (41) | 0.817 |
| Coronary angiogram | 89.7 (52) | 78.6 (33) | 0.126 |
| Correct diagnosis | 87.9 (51) | 69.0 (29) | 0.020 |
| Treatment: aspirin | 34.5 (20) | 26.2 (11) | 0.376 |
| Treatment: heparin | 36.2 (21) | 21.4 (9) | 0.111 |
| Treatment: nitrates | 50.0 (29) | 38.1 (16) | 0.238 |
| Management: max. 11 points | 8.1 ± 0.2 | 7.0 ± 0.2 | 0.001 |
| Total score: max. 19 points | 12.0 ± 0.3 | 10.7 ± 0.3 | 0.004 |

**
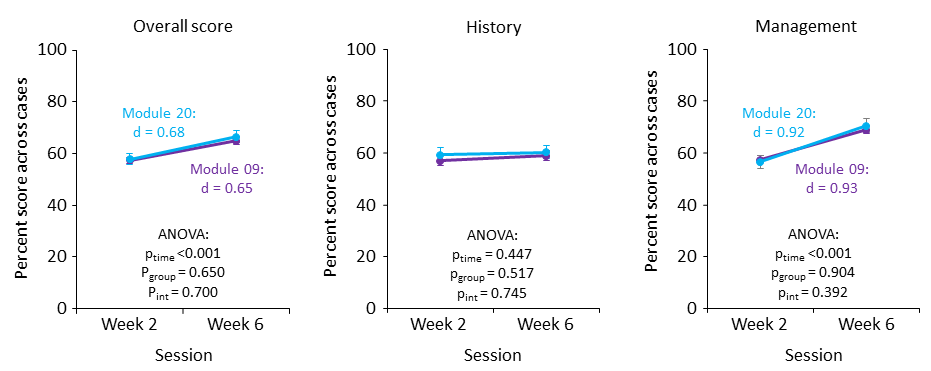
eFigure 1: Change in total, history and management scores from week 2 to week 6 in both modules (data collection in summer term 2017).** Error bars indicate standard errors of the mean. p_int_, p for interaction (ANOVA)
